# Antiretroviral treatment interruption among people living with HIV during COVID‐19 outbreak in China: a nationwide cross‐sectional study

**DOI:** 10.1002/jia2.25637

**Published:** 2020-11-01

**Authors:** Yinghui Sun, Hui Li, Ganfeng Luo, Xiaojun Meng, Wei Guo, Thomas Fitzpatrick, Yunlong Ao, Anping Feng, Bowen Liang, Yuewei Zhan, Amakobe Sande, Feng Xie, Ying Wang, Han‐Zhu Qian, Yong Cai, Huachun Zou

**Affiliations:** ^1^ School of Public Health (Shenzhen) Sun Yat‐sen University Shenzhen China; ^2^ Shizhong District Center for Disease Control and Prevention Jinan China; ^3^ Wuxi Municipal Center for Disease Control and Prevention Wuxi China; ^4^ UNAIDS China Office Beijing China; ^5^ Department of Internal Medicine University of Washington Seattle WA USA; ^6^ Department of Infectious Diseases Guangzhou Eighth People’s Hospital affiliated to Guangzhou Medical School Guangzhou China; ^7^ Chongqing Blue Sky nongovernment organization Chongqing China; ^8^ School of Public Health Shanghai Jiao Tong University School of Medicine Shanghai China; ^9^ SJTU‐Yale Joint Center for Biostatistics and Data Science Department of Bioinformatics and Biostatistics School of Life Science and Biotechnology Shanghai Jiao Tong University (SJTU) Shanghai China; ^10^ Yale School of Public Health New Haven CT USA; ^11^ Shenzhen Center for Disease Control and Prevention Shenzhen China; ^12^ Kirby Institute University of New South Wales Sydney Australia

**Keywords:** antiretroviral therapy interruption, antiretroviral therapy, HIV, lockdown, COVID‐19, China

## Abstract

**Introduction:**

Social disruption associated with coronavirus disease 2019 (COVID‐19) threatens to impede access to regular healthcare, including for people living with HIV (PLHIV), potentially resulting in antiretroviral therapy (ART) interruption (ATI). We aimed to explore the characteristics and factors associated with ATI during the COVID‐19 outbreak in China.

**Methods:**

We conducted an online survey among PLHIV by convenience sampling through social media between 5 and 17 February 2020. Respondents were asked to report whether they were at risk of ATI (i.e. experienced ATI, risk of imminent ATI, threatened but resolved risk of ATI [obtaining ART prior to interruption]) or were not at risk of ATI associated with the COVID‐19 outbreak. PLHIV were also asked to report perceived risk factors for ATI and sources of additional ART. The factors associated with the risk of ATI were assessed using logistic regression. We also evaluated the factors associated with experienced ATI.

**Results:**

A total of 5084 PLHIV from 31 provinces, autonomous regions and municipalities in mainland China completed the survey, with valid response rate of 99.4%. The median age was 31 years (IQR 27 to 37), 96.5% of participants were men, and 71.3% were men who had sex with men. Over one‐third (35.1%, 1782/5084) reported any risk of ATI during the COVID‐19 outbreak, including 2.7% (135/5084) who experienced ATI, 18.0% (917/5084) at risk of imminent ATI and 14.4% (730/5084) at threatened but resolved risk. PLHIV with ATI were more likely to have previous interruptions in ART (aOR 8.3, 95% CI 5.6 to 12.3), travelled away from where they typically receive HIV care (aOR 3.0, 95% CI 2.1 to 4.5), stayed in an area that implemented citywide lockdowns or travel restrictions to control COVID‐19 (aOR 2.5, 95% CI 1.4 to 4.6), and be in permanent residence in a rural area (aOR 3.7, 95% CI 2.3 to 5.8).

**Conclusions:**

A significant proportion of PLHIV in China are at risk of ATI during the COVID‐19 outbreak and some have already experienced ATI. Correlates of ATI and self‐reported barriers to ART suggest that social disruptions from COVID‐19 have contributed to ATI. Our findings demonstrate an urgent need for policies and interventions to maintain access to HIV care during public health emergencies.

## 1 Introduction

A cluster of pneumonia cases of unknown cause appeared in Wuhan, China in December 2019. A novel coronavirus, SARS‐CoV‐2, was soon identified as the cause, and by February 2020 the number of confirmed cases of coronavirus disease 2019 (COVID‐19) in China approached 80,000 (about 67,000 in Hubei province) [1]. National and regional government enacted a series of prevention and control measures aimed at COVID‐19. Many large metropolitan areas in 28 provinces, autonomous regions and municipalities across China instituted citywide lockdowns on 30 January under which all non‐essential shops were closed, public transportation suspended and residents confined to their homes [2, 3]. Travel restrictions were also adopted, severely limiting air and train travel, as well as postal and delivery services.

While deemed necessary to contain the growing COVID‐19 outbreak, these measures profoundly impacted daily life in China, including access to primary healthcare and prescription medications. Anticipating social disruption from COVID‐19 would complicate the management of chronic diseases, professional societies and experts published recommendations on how to best care for patients with cardiovascular disease, diabetes and cancer during the outbreak [4, 5, 6, 7]. People living with HIV (PLHIV) in China were also at risk of experiencing disruptions in care, particularly because HIV care and antiretroviral therapy (ART) in China are nearly exclusively provided through the government‐designated clinics and hospitals of their household registration. According to the specific requirements of different regions, PLHIV could obtain one month or three months of ART at a time [8, 9]. Recognizing that access to ART may be hampered, especially for those quarantined away from home, the Chinese Center for AIDS/STD Control and Prevention issued a nationwide directive on 26 January 2020 to relax restrictions on where PLHIV could obtain ART [10]. Under this directive, PLHIV could obtain one month of ART from any local HIV care clinic or hospital.

Despite efforts by the government agencies and professional societies to mitigate the impact of COVID‐19, many PLHIV have likely experienced some disruption in primary HIV care, including ART interruption (ATI). These disruptions may negatively impact the health of PLHIV as ATI is associated with both increased likelihood of opportunistic infections, more severe illnesses, HIV‐associated mortality and HIV transmission [11, 12]. Initial reports suggest as many as one‐third of PLHIV in China may be at risk of experiencing ATI during the outbreak [13], however, there are few available data describing which PLHIV are most at risk and which adaptive strategies have been used to avoid ATI. This study aims to better characterize the extent of and important correlates with ATI in China during the COVID‐19 outbreak.

## 2 Methods

### 2.1 Study design

We conducted a nationwide cross‐sectional online survey of PLHIV in China between 5 February and 17 February 2020. Individuals were recruited by convenience sampling through social media accounts of popular HIV‐themed WeChat public accounts. WeChat is a multi‐purpose social media and messaging platform in China with over 1 billion monthly active users [14]. Many PLHIV in China subscribed to HIV‐themed WeChat public accounts to receive and exchange information. An advertisement with a link to the online survey was promoted through WeChat public accounts. We initially posted study information in 12 WeChat public groups (with approximately 500 members each) from various regions in the country and encouraged them to repost further, including Li Hui Shi Kong, an account with over 76,000 PLHIV subscribers. As such up to 100,000 PLHIV would have noticed our recruitment information.

All participants read a consent form and selected ‘agree’ before beginning the survey hosted on the online questionnaire platform Wenjuanxing (www.wjx.com). The survey was first screened for eligibility. Eligible participants were at least 18‐years‐old, had a known diagnosis of HIV, and were currently on ART. We excluded those who did not provide informed consent. A preliminary survey of 15 PLHIV was conducted to modify questionnaire content before formal recruitment. Recruitment was stopped the day when the total number of participants exceeded 5000.

### 2.2 Measures

The primary outcome of interest was ATI during the COVID‐19 outbreak. We defined ATI as missing one or more days of ART. Participants were asked to self‐report whether they had experienced ATI of any duration after 10 January 2020 as well as whether they continued to be off ART at the time of survey completion. Participants were also asked to report their self‐assessed risk of ATI, which included (1) having experienced ATI, (2) nearly experiencing ATI but obtaining ART prior to interruption (i.e. threatened but resolved risk of ATI), (3) having fewer than 10 days of antiretroviral (ARV) drugs on hand without a clear way to obtain or refill antiretroviral medications (i.e. risk of imminent ATI) and (4) no risk of ATI.

The survey instrument also asked about potential correlates of ATI. We collected sociodemographic information (sex, age, highest level of education, occupation, income, permanent residence) and HIV treatment history (route of HIV acquisition, age at HIV diagnosis, age at ART initiation, prior ATI, date of most recent CD4 cell count and HIV viral load, site of primary HIV care and source of ART prior to the COVID‐19 outbreak). The survey also asked where participants lived and travelled during the COVID‐19 outbreak and whether they had been impacted by certain COVID‐19 prevention and control measures, including citywide lockdowns and travel restrictions. Participants were asked to report strategies they employed to address threatened or experienced ATI, sources from which they attempted to obtain additional ART and knowledge of the government policies regarding ART access during the COVID‐19 outbreak. For this study time period of the COVID‐19 outbreak was defined as any time after 10 January 2020, which was the beginning of Chinese New Year holiday when hundreds of millions of people usually travel back to hometown. For PLHIV, this may have meant that they would temporarily leave the city where they lived and usually obtained ART from their HIV care clinic or hospital.

### 2.3 Analysis

Descriptive statistical analysis was applied to summarize experiences with ATI as well as sociodemographic information and potential correlates of ATI. Differences between PLHIV at risk of ATI (either experienced, risk of imminent or threatened but resolved ATI) and PLHIV without risk of ATI were compared using Pearson’s Chi‐squared test. The associations between risk of ATI and correlates were analysed using multivariate logistic regression that adjusted for potential confounders selected a priori as well as significant correlates of risk of ATI. Similarly, associations between PLHIV who did and did not experience ATI were analysed using multivariate logistic regression. Results were reported as adjusted ORs (aOR) with corresponding 95% confidence intervals (95% CI). Multicollinearity of variables included in the multivariate logistic regression model was tested with a variance inflation factor (VIF) [15]. The final model had a mean VIF of 2.7 in risk of ATI and 2.7 in experienced ATI. Statistical analysis was conducted using Stata software 15.0 (StataCorp LLC, College Station, TX).

Chordal graphs were generated to present the movement of PLHIV between provinces in the leadup to the COVID‐19 outbreak, using R software 3.6.0 (R Core Team, Vienna, Austria). Bar graphs summarizing sources of ARV drugs were plotted using Excel software 2016 (Microsoft, Washington, USA).

### 2.4 Ethics

This study was conducted with the approval of the Sun Yat‐sen University Ethics Committee (SYSU‐SPH2020008).

## 3 Results

### 3.1 Participant characteristics

A total of 5084 PLHIV completed the online survey. The median age of respondents was 31 years (interquartile range [IQR] 27 to 37 years). Most participants (96.5%) were men, and over two‐thirds (71.3%) were men who have sex with men (MSM). The median time since HIV diagnosis and ART initiation were 2.7 years (IQR 1.3 to 4.4 years) and 2.3 years (IQR 1.1 to 3.9 years) respectively. The majority (89.8%) received free government‐sponsored ART, whereas few (5.3%) purchased ART out‐of‐pocket. Most participants (77.6%) reported their last viral load was undetectable. Sociodemographic characteristics and HIV care of participants are summarized in Table 1.

**Table 1:** Demographic characteristics and HIV care among PLHIV in China responding to a nationwide online survey during the COVID‐19 outbreak

Characteristics	Total (N = 5084) n (%)	No risk of ATI (N = 3302) n (%)	Threatened but resolved risk of ATI (N = 730) n (%)	Risk of imminent ATI (N = 917) n (%)	Experienced ATI (N = 135) n (%)	*P* value
Sex						0.526
Male	4904 (96.46)	3179 (96.27)	711 (97.40)	884 (96.40)	130 (96.30)	
Female	180 (3.54)	123 (3.73)	19 (2.60)	33 (3.60)	5 (3.70)	
Age, year						**<0.001**
18 to 29	2092 (41.15)	1267 (38.37)	336 (46.03)	417 (45.47)	72 (53.33)	
30 to 39	2063 (40.58)	1376 (41.67)	289 (39.59)	349 (38.06)	49 (36.30)	
≥40	929 (18.27)	659 (19.96)	105 (14.38)	151 (16.47)	14 (10.37)	
Education						**<0.001**
College or above	3647 (71.73)	2366 (71.65)	583 (79.86)	606 (66.09)	92 (68.15)	
High school or below	1437 (28.27)	936 (28.35)	147 (20.14)	311 (33.91)	43 (31.85)	
Occupation						**<0.001**
Student	364 (7.16)	193 (5.84)	59 (8.08)	102 (11.12)	10 (7.41)	
Farmer	218 (4.29)	126 (3.82)	26 (3.56)	57 (6.22)	9 (6.67)	
Employed	4073 (80.11)	2674 (80.98)	610 (83.56)	683 (74.48)	106 (78.52)	
Unemployed	429 (8.44)	309 (9.36)	35 (4.79)	75 (8.18)	10 (7.41)	
Salary (RMB)						**<0.001**
<2000	923 (18.15)	565 (17.11)	112 (15.34)	217 (23.66)	29 (21.48)	
2000 to 4999	1920 (37.77)	1285 (38.92)	234 (32.05)	338 (36.86)	63 (46.67)	
5000 to 9999	1553 (30.55)	999 (30.25)	253 (34.66)	267 (29.12)	34 (25.19)	
≥10000	688 (13.53)	453 (13.72)	131 (17.95)	95 (10.36)	9 (6.67)	
HIV transmission route						**0.010**
Male‐male sex	3627 (71.34)	2379 (72.05)	542 (74.25)	607 (66.19)	99 (73.33)	
Male‐female sex	499 (9.82)	315 (9.54)	65 (8.90)	109 (11.89)	10 (7.41)	
Other or not sure	958 (18.84)	608 (18.41)	123 (16.85)	201 (21.92)	26 (19.26)	
CD4 cell count (per ul) at HIV diagnosis^a^						0.057
<200	1103 (21.73)	753 (22.84)	135 (18.52)	187 (20.41)	28 (20.74)	
200 to 349	1574 (31.00)	1022 (31.00)	225 (30.86)	294 (32.10)	33 (24.44)	
350 to 499	1305 (25.70)	813 (24.66)	198 (27.16)	248 (27.07)	46 (34.07)	
≥500	1095 (21.57)	709 (21.50)	171 (23.46)	187 (20.41)	28 (20.74)	
Viral load at last test						**0.001**
Undetectable	3946 (77.62)	2599 (78.71)	583 (79.86)	672 (73.28)	92 (68.15)	
Detectable	344 (6.77)	211 (6.39)	42 (5.75)	78 (8.51)	13 (9.63)	
Not sure	794 (15.62)	492 (14.90)	105 (14.38)	167 (18.21)	30 (22.22)	
Source of ART						**0.001**
Free government‐sponsored ART	4567 (89.83)	2940 (89.04)	647 (88.63)	855 (93.24)	125 (92.59)	
Purchase out of pocket	268 (5.27)	201 (6.09)	40 (5.48)	25 (2.73)	2 (1.48)	
Both	249 (4.90)	161 (4.88)	43 (5.89)	37 (4.03)	8 (5.93)	
Ever obtained ART via postage						**<0.001**
Yes	1542 (30.33)	909 (27.53)	345 (47.26)	238 (25.95)	50 (37.04)	
No	3542 (69.67)	2393 (72.47)	385 (52.74)	679 (74.05)	85 (62.96)	

RMB 100 ≈ USD 14. The bold values are statistically significant (*P* < 0.05) in Pearson’s Chi‐squared test. HIV transmission reclassified for multiple choices of “Your route of HIV transmission”, HIV transmission of “intravenous drug use”, “Former commercial blood donors”, “Mother‐to‐child”, “Other”, “Not sure” classified as “Other or not sure”. ART, antiretroviral therapy; ATI, antiretroviral therapy interruption; PLHIV, people living with HIV.

^a^ 7 missing.

### 3.2 ATI during the COVID‐19 outbreak

Over one‐third (35.1%, 1782/5084) reported any risk of ATI during the COVID‐19 outbreak, including 2.7% (135/5084) who experienced ATI of any duration, 18.0% (917/5084) who were at risk of imminent ATI and 14.4% (730/5084) who had threatened but resolved risk of ATI. Of the 135 participants who experienced ATI during this period, 50.4% (68/135) and 49.6% (67/135) had resolved or continued to experience ATI at the time of survey completion respectively. The median duration of ATI was 3 days (IQR 1 to 6 days).

The geographical distribution of risk of ATI by province is presented in Table 2. The highest rate of experienced, threatened or imminent ATI, as a single endpoint, was found in Hubei (58.7%), followed by Xinjiang (51.8%) and Zhejiang (51.5%) Provinces, whereas the highest rate of experienced ATI occurred in Xinjiang (7.9%), followed by Gansu (5.9%) and Henan (5.0%) Provinces.

**Table 2:** Proportion of PLHIV at risk of ATI and PLHIV who flowed in and out of each province during the COVID‐19 outbreak in 31 provinces and municipalities in mainland China

Province	Region in China	No. PLHIV recruited	% PLHIV at risk of ATI	% PLHIV at threatened but resolved risk of ATI	% PLHIV at risk of imminent ATI	% PLHIV who experienced ATI	No. PLHIV flowed out to another province	No. PLHIV flowed in from another province
Jiangsu	Eastern	425	33.41	15.29	16.94	1.18	120	57
Shanghai		154	35.72	20.13	12.99	2.60	82	21
Shandong		256	30.85	15.23	14.06	1.56	38	72
Jiangxi		93	34.41	11.83	19.35	3.23	23	51
Fujian		99	26.26	10.10	14.14	2.02	15	40
Anhui		132	36.36	16.67	17.42	2.27	25	93
Zhejiang		371	51.48	23.18	23.72	4.58	143	29
Hubei	South‐Central	223	58.74	18.83	37.67	2.24	36	141
Hunan		171	28.66	11.70	12.87	4.09	31	78
Henan		238	44.54	17.23	22.27	5.04	25	106
Hainan		51	21.56	9.80	11.76	0.00	14	22
Guangdong		507	38.46	12.23	23.27	2.96	239	34
Guangxi		98	29.59	9.18	18.37	2.04	14	45
Chongqing	Southwest	208	30.77	15.87	12.50	2.40	39	26
Sichuan		314	27.71	12.42	13.06	2.23	57	84
Yunnan		94	27.66	8.51	17.02	2.13	26	18
Guizhou		167	25.15	7.19	14.37	3.59	15	33
Xinjiang	Northwest	139	51.79	21.58	22.30	7.91	20	25
Shaanxi		185	38.37	14.05	21.08	3.24	36	34
Gansu		51	37.25	11.76	19.61	5.88	4	29
Heilongjiang	Northeast	104	24.03	8.65	12.50	2.88	15	48
Jilin		77	20.78	9.09	10.39	1.30	18	28
Liaoning		187	19.78	8.02	11.23	0.53	31	32
Inner Mongolia		79	26.58	11.39	15.19	0.00	3	28
Beijing	North	310	33.23	16.45	15.81	0.97	225	12
Tianjin		85	27.06	8.24	15.29	3.53	23	17
Hebei		117	29.91	11.11	16.24	2.56	18	83
Shanxi		67	22.39	8.96	13.43	0.00	4	49

Provinces that had less than 30 participants were not included in this analysis, including Tibet (3), Qinghai (9) and Ningxia (19). Participants resided in foreign areas (49) were not shown. ATI, antiretroviral therapy interruption; PLHIV, people living with HIV.

Among all participants, the most commonly perceived risk factors for experiencing ATI during the COVID‐19 outbreak included restrictions on movement as a consequence of lockdown and traffic restrictions (83.3%), insufficient ART reserve at home (71.4%) and suspension of postal and delivery services (50.6%) (Table 3). Among the 1782 participants who reported experiencing or being at risk of ATI, the most common self‐reported barrier to obtaining ART was fear of disclosing HIV status to others by seeking refills of ARV drugs during the COVID‐19 outbreak (77.4%). Other frequently reported barriers included inability to travel to obtain ARV drugs because of lockdown and traffic restrictions (65.8%) and cumbersome administrative procedures to obtain ARV drugs from clinics or hospitals other than one’s site of primary HIV care (51.1%). The frequency of self‐reported barriers to obtaining ARV drugs was similar between participants who experienced ATI, were at risk of imminent ATI, and had threatened but resolved the risk of ATI (Table 4).

**Table 3:** Behaviors and risk factors comparing PLHIV with and without risk of ATI during the COVID‐19 outbreak in China

Characteristics	Total (N=5084) n (%)	No risk of ATI (N=3302) n (%)	Threatened but resolved risk of ATI (N=730) n (%)	Risk of imminent ATI (N=917) n (%)	Experienced ATI (N=135) n (%)	*p* value
Previous ATI before COVID‐19						
Yes	1070 (21.05)	600 (18.17)	190 (26.03)	193 (21.05)	87 (64.44)	**<0.001**
No	4014 (78.95)	2702 (81.83)	540 (73.97)	724 (78.95)	48 (35.56)	
Reason for previous ATI						
Could not obtain ART in time	232 (21.68)	77 (12.83)	51 (26.84)	57 (29.53)	47 (54.02)	**<0.001**
Decided to stop ART	55 (5.14)	36 (6.00)	5 (2.63)	8 (4.15)	6 (6.90)	0.230
Intolerable ART side effects	173 (16.17)	99 (16.50)	28 (14.74)	30 (15.54)	16 (18.39)	0.871
Forgot to take ART	738 (68.97)	445 (74.17)	134 (70.53)	123 (63.73)	36 (41.38)	**<0.001**
Village/street/community on lockdown during COVID‐19						
Yes	3582 (70.46)	2096 (63.48)	569 (77.95)	800 (87.24)	117 (86.67)	**<0.001**
No	1281 (25.20)	1036 (31.37)	140 (19.18)	91 (9.92)	14 (10.37)	
Not sure	221 (4.35)	170 (5.15)	21 (2.88)	26 (2.84)	4 (2.96)	
Traveled away from site of primary HIV care during COVID‐19						
Yes	1967 (38.69)	914 (27.68)	448 (61.37)	532 (58.02)	73 (54.07)	**<0.001**
No	3117 (61.31)	2388 (72.32)	282 (38.63)	385 (41.98)	62 (45.93)	
No. days of planned travel^a^						
<15	1124 (57.23)	525 (57.57)	290 (64.88)	255 (47.93)	54 (73.97)	**<0.001**
15 to 29	500 (25.46)	191 (20.94)	113 (25.28)	182 (34.21)	14 (19.18)	
≥30	340 (17.31)	196 (21.49)	44 (9.84)	95 (17.86)	5 (6.85)	
No. days of pills prepared before travel^b^						
<15	378 (19.24)	179 (19.63)	122 (27.23)	49 (9.21)	28 (38.36)	**<0.001**
15 to 29	607 (30.89)	192 (21.05)	192 (42.86)	184 (34.59)	39 (53.42)	
≥30	980 (49.87)	541 (59.32)	134 (29.91)	299 (56.20)	6 (8.22)	
Location during CNY 2020 (January 10 to February 17)						
Domestic urban area	2792 (54.92)	2101 (63.63)	337 (46.16)	320 (34.90)	34 (25.19)	**<0.001**
Domestic rural area	1787 (35.15)	857 (25.95)	348 (47.67)	503 (54.85)	79 (58.52)	
Overseas	132 (2.60)	95 (2.88)	19 (2.60)	14 (1.53)	4 (2.96)	
Unknown^c^	373 (7.34)	249 (7.54)	26 (3.56)	80 (8.72)	18 (13.33)	
Perceived risk factors for ATI						
Lockdown and travel restrictions	4235 (83.30)	2651 (80.28)	662 (90.68)	804 (87.68)	118 (87.41)	**<0.001**
Insufficient ART supply at HIV clinic or pharmacy	2402 (47.25)	1718 (52.03)	340 (46.58)	302 (32.93)	42 (31.11)	**<0.001**
Suspension of postal and courier services	2570 (50.55)	1541 (46.67)	466 (63.84)	492 (53.65)	71 (52.59)	**<0.001**
Insufficient ART reserve at home	3630 (71.40)	2279 (69.02)	585 (80.14)	671 (73.17)	95 (70.37)	**<0.001**
Borrowed pills from other PLHIV before COVID‐19						
Yes	136 (58.62)	39 (50.65)	40 (78.43)	32 (56.14)	25 (53.19)	**0.012**
No	96 (41.38)	38 (49.35)	11 (21.57)	25 (43.86)	22 (46.81)	

The bold values are statistically significant (*p* < 0.05) in Pearson’s Chi squared test. ART, antiretroviral therapy; ATI, antiretroviral therapy interruption; CNY, Chinese New Year; PLHIV, people living with HIV.

^a^ 3 missing

^b^ 2 missing

^c^ the location cannot be judged based on the information provided, shown as “unknown”, not discussed.

**Table 4:** Barriers to obtain ART and strategies to solve ATI among PLHIV at risk of ATI during the COVID‐19 outbreak in China

Characteristics	Total (N = 1782) N (%)	Threatened but resolved risk of ATI (N = 730) n (%)	Risk of imminent ATI (N = 917) n (%)	Experienced ATI (N = 135) n (%)
No. days of ART reserve when PLHIV realized risk of ATI: Median (IQR)	10 (6 to 15)	10 (6 to 20)	10 (7 to 15)^a^	5 (0 to 14)^b^
Strategies used to solve ATI				
Disclosed HIV status and applied for permission to travel to an HIV clinic	87 (4.88)	42 (5.75)	42 (4.58)	3 (2.22)
Pretended to have a disease other than HIV requiring urgent care	862 (48.37)	436 (59.73)	374 (40.79)	52 (38.52)
Decided not to obtain ART to avoid disclosure of HIV status	637 (35.75)	80 (10.96)	502 (54.74)	55 (40.74)
Other^c^	361 (20.26)	199 (27.26)	136 (14.83)	26 (19.26)
Barriers to obtaining ART from HIV clinics during COVID‐19				
Cumbersome administrative procedures	911 (51.12)	379 (51.92)	463 (50.49)	69 (51.11)
Lockdown and travel restrictions	1172 (65.77)	449 (61.51)	627 (68.38)	96 (71.11)
Fear of disclosing HIV status	1379 (77.38)	584 (80.00)	696 (75.90)	99 (73.33)
Other^d^	192 (10.77)	90 (12.33)	86 (9.38)	16 (11.85)
Preferred way of obtaining additional ART during COVID‐19				
Mailed by one’s primary HIV clinic	1418 (79.57)	580 (79.45)	731 (79.72)	107 (79.26)
Dispensed by a nearby HIV clinic other than one’s primary HIV clinic	251 (14.09)	100 (13.70)	128 (13.96)	23 (17.04)
Purchased online and delivered by mail	113 (6.34)	50 (6.85)	58 (6.32)	5 (3.70)

ART, antiretroviral therapy; ATI, antiretroviral therapy interruption; IQR, interquartile range; PLHIV, people living with HIV.

^a^ 1 missing

^b^ 1 missing

^c^ other includes violating travel restrictions to reach an HIV clinic and purchasing ART out of pocket

^d^ other includes insufficient ART supply at local clinics, suspension of postal and courier services and no response from local clinics.

**Figure 1:**
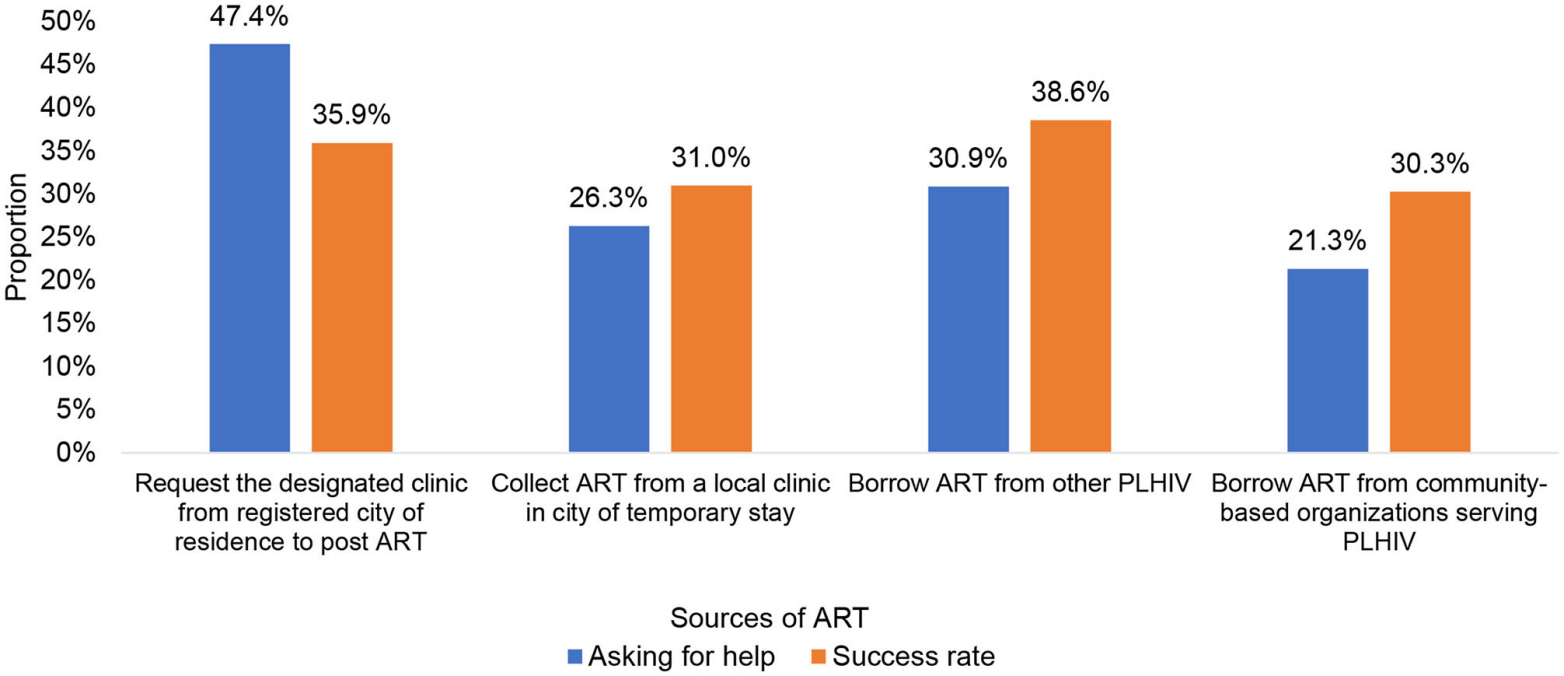
Sources of ART among PLHIV at risk of ATI in China during COVID‐19 outbreak. The X‐axis is a source of ART. The Y‐axis is the proportion of PLHIV who asked for help with ART reservation and the proportion of PLHIV who successfully obtained ART. ART, antiretroviral therapy; PLHIV, people living with HIV; ATI, antiretroviral therapy interruption.

The most common strategies used to address experienced, threatened or imminent ATI included asking one’s primary HIV clinic to post‐additional ART (47.4%), attempting to borrow ART from other PLHIV (30.9%) and attempting to borrow ART from community‐based organizations (CBOs) serving PLHIV (21.3%). The success rate of obtaining additional ART through these routes was 35.9%, 38.6% and 30.3% respectively (Figure 1and Table S1). When faced with imminent ATI, nearly half of the participants (48.4%) obtained urgent healthcare evaluation by pretending to have a disease other than HIV. Few participants disclosed their HIV status and applied for permission to travel to their site of primary HIV care (4.9%) (Table 4).

### 3.3 ATI prior to the COVID‐19 outbreak

Over one in every five participants (21.1%) had experienced at least one episode of ATI prior to the COVID‐19 outbreak. The most common reported reasons for previous ATI prior to the COVID‐19 outbreak included forgetting to take ART (69.0%), being unable to obtain ART in time (21.7%) and intolerable ART side effects (16.2%) (Table 3).

### 3.4 Travel and ART reserve during the COVID‐19 outbreak

**Figure 2:**
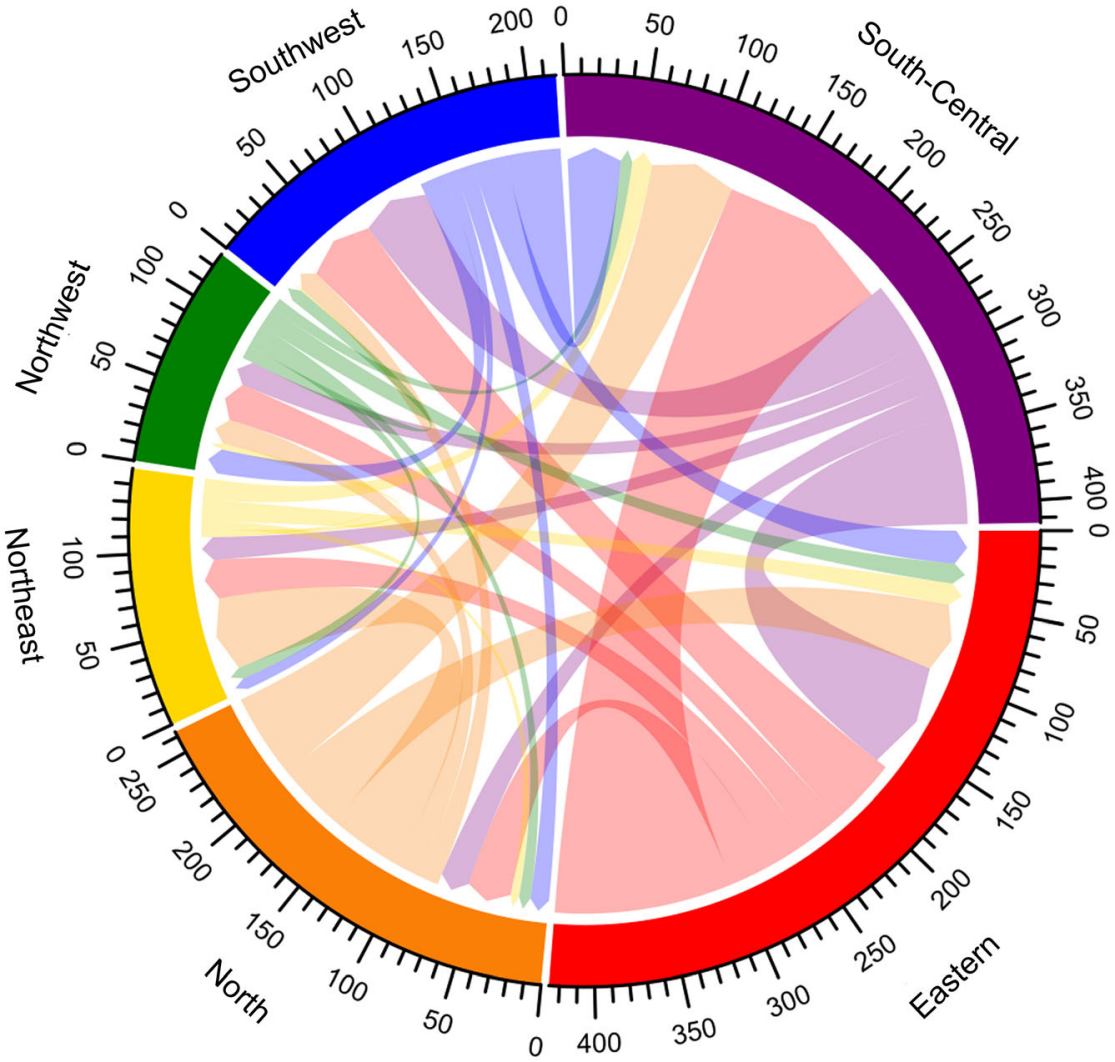
Chordal graph of flow of PLHIV between regions during the COVID‐19 outbreak (unit: person). A chord stands for a migration flow. The arrow from the origin to the destination indicates the flow direction. The width of the chord represents the flux of the flow. Each scale corresponds to 10 individuals in the graph. Six different colour represent six regions in 31 provinces and municipalities in mainland China. Red, purple, blue, green, yellow and orange represent Eastern, South‐Central, Southwest, Northwest, Northeast and North China respectively. PLHIV, people living with HIV.

**Figure 3:**
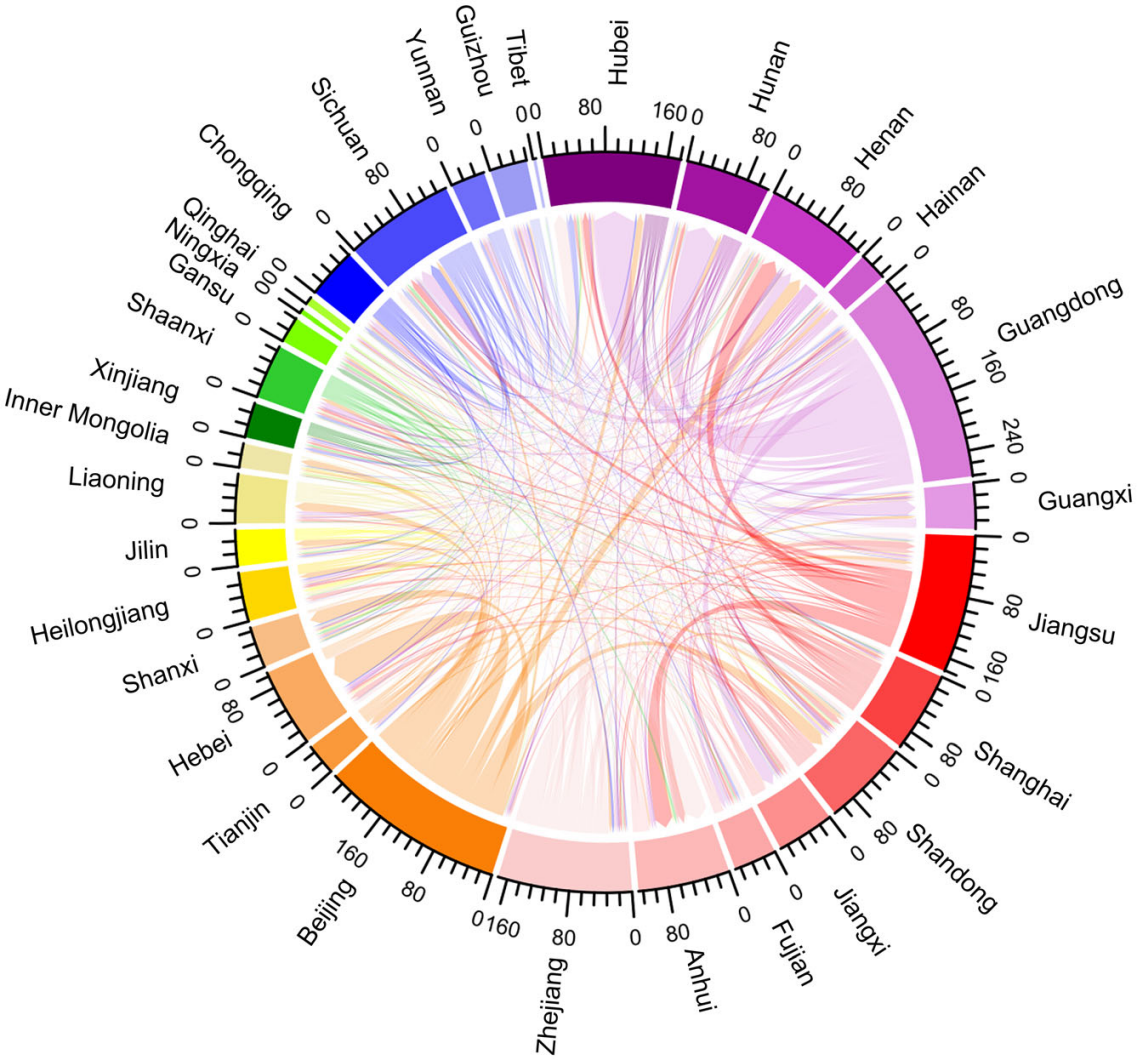
Chordal graph of flow of PLHIV between provinces during the COVID‐19 outbreak (unit: person). A chord stands for a migration flow. The arrow from the origin to the destination indicates the flow direction. The width of the chord represents the flux of the flow. Each scale corresponds to 16 individuals in the graph. Six different colour series represent six regions in 31 provinces and municipalities in mainland China. Red, purple, blue, green, yellow and orange series represent Eastern (Jiangsu, Shanghai, Shandong, Jiangxi, Fujian, Anhui, Zhejiang), South‐Central (Hubei, Hunan, Henan Hainan, Guangdong, Guangxi), Southwest (Chongqing, Sichuan, Yunnan, Guizhou, Tibet), Northwest (Xinjiang, Shaanxi, Gansu, Ningxia, Qinghai), Northeast (Heilongjiang, Jilin, Liaoning, Inner Mongolia) and North (Beijing, Tianjin, Hebei, Shanxi) China respectively. PLHIV, people living with HIV.

Over two‐thirds of participants (70.5%, 3582/5084) experienced a citywide or village‐wide lockdown and 38.7% (1967/5084) travelled away from their site of primary HIV care during the COVID‐19 outbreak. Travel patterns are shown in Figures 2 and 3. More PLHIV in Eastern and North flowed out, especially for Zhejiang and Beijing provinces; more PLHIV flowed into South‐Central, especially for Hubei and Henan provinces. Among the 1967 participants who travelled during the COVID‐19 outbreak, 57.2% planned to travel for less than 15 days, 49.9% travelled with enough ARV drugs to last more than one month and 28.6% reported being stranded in a location away from their primary residence longer than anticipated because of lockdown measures or travel restrictions. Only 1.9% did not carry enough pills for the number of days they originally planned to travel.

### 3.5 Correlates of ATI and risk of ATI

In multivariate regression analysis, PLHIV who experienced ATI during the COVID‐19 outbreak were more likely to have previous interruptions in ART (aOR 8.3, 95% CI 5.6 to 12.3), travelled away from where they typically receive HIV care (aOR 3.0, 2.1 to 4.5), stayed in an area that implemented citywide lockdown and travel restrictions (aOR 2.5, 1.4 to 4.6) and permanent residence in a rural area (aOR 3.7, 2.3 to 5.8). Self‐reported risk of ATI was associated with similar correlates, including previous interruptions in ART (aOR 1.6, 1.3 to 1.8), travel away from where one typically receives HIV care (aOR 3.3, 2.9 to 3.8), rural residence (aOR 2.3, 2.0 to 2.7) and living in an area that implemented citywide lockdown and travel restriction (aOR 2.4, 2.0 to 2.8). The risk of ATI was also positively correlated with relying on ART to be delivered by post (aOR 1.4, 1.2 to 1.6) and negatively associated with buying ART out of pocket (aOR 0.7, 0.5 to 0.9) (Table 5).

**Table 5:** Correlates of experienced ATI and risk of ATI among PLHIV during COVID‐19 outbreak in China

Characteristics	Experienced ATI		Risk of ATI	
	aOR (95% CI)	*P* value	aOR (95% CI)	*P* value
Age, year				
18 to 29	1.51 (0.79 to 2.89)	0.215	1.08 (0.89 to 1.32)	0.416
30 to 39	1.21 (0.63 to 2.31)	0.573	1.01 (0.84 to 1.22)	0.913
≥40	Ref.		Ref.	
Occupation				
Student	0.92 (0.34 to 2.50)	0.869	**1.55 (1.11 to 2.17)**	**0.011**
Farmer	1.98 (0.71 to 5.51)	0.189	**1.70 (1.17 to 2.48)**	**0.006**
Employed	1.62 (0.70 to 3.73)	0.262	**1.39 (1.03 to 1.87)**	**0.033**
Unemployed	Ref.		Ref.	
Salary (RMB)				
<2000	Ref.		Ref.	
2000 to 4999	0.93 (0.48 to 1.80)	0.834	0.86 (0.67 to 1.11)	0.246
5000 to 9999	0.84 (0.40 to 1.75)	0.636	1.05 (0.81 to 1.38)	0.701
≥10000	0.50 (0.19 to 1.31)	0.157	1.09 (0.81 to 1.47)	0.578
CD4 cell count (per ul) at HIV diagnosis^a^				
＜200	Ref.		Ref.	
200 to 349	0.68 (0.39 to 1.19)	0.176	1.02 (0.85 to 1.22)	0.838
350 to 499	1.20 (0.71 to 2.04)	0.495	1.20 (0.99 to 1.44)	0.060
≥500	0.71 (0.39 to 1.29)	0.260	1.05 (0.87 to 1.28)	0.600
Viral load at last test				
Undetectable	Ref.		Ref.	
Detectable	1.70 (0.87 to 3.35)	0.123	1.19 (0.92 to 1.53)	0.176
Not sure	1.55 (0.96 to 2.50)	0.071	1.08 (0.90 to 1.29)	0.390
Source of ART				
Free government‐sponsored ART	Ref.		Ref.	
Purchase out of pocket	0.36 (0.08 to 1.54)	0.167	**0.67 (0.49 to 0.92)**	**0.014**
Both	1.52 (0.66 to 3.47)	0.321	1.06 (0.78 to 1.44)	0.706
Previous ATI before COVID‐19				
Yes	**8.31 (5.64 to 12.26)**	**<0.001**	**1.55 (1.33 to 1.80)**	**<0.001**
No	Ref.		Ref.	
Ever obtained ART via postage				
Yes	1.37 (0.92 to 2.04)	0.125	**1.40 (1.22 to 1.62)**	**<0.001**
No	Ref.		Ref.	
Travelled away from site of primary HIV care				
Yes	**3.02 (2.05 to 4.47)**	**<0.001**	**3.33 (2.92 to 3.79)**	**<0.001**
No	Ref.		Ref.	
Location during CNY 2020 (Jan 10‐Feb 17)				
Domestic city	Ref.		Ref.	
Domestic rural	**3.66 (2.32 to 5.78)**	**<0.001**	**2.31 (2.01 to 2.67)**	**<0.001**
Overseas	2.41 (0.80 to 7.29)	0.120	1.06 (0.71 to 1.61)	0.766
Unknown	**3.70 (1.97 to 6.97)**	**<0.001**	**1.47 (1.14 to 1.88)**	**0.003**
Village/street/community on lockdown				
Yes	**2.53 (1.39 to 4.62)**	**0.002**	**2.37 (2.00 to 2.81)**	**<0.001**
No	Ref.		Ref.	
Not sure	1.13 (0.35 to 3.67)	0.835	1.21 (0.84 to 1.74)	0.307

RMB 100 ≈ USD 14. The bold values are statistically significant (*P* < 0.05). The observations of “No. days of pills prepared before travel” were 1964, far less than total sample (5077, missing 7, less than 0.1%), so this was not included in the multivariable logistic regression analysis. The observations in the “experienced ATI” model and “risk of ATI” models were 3432 (7 missing) and 5077 (7 missing) respectively. aOR, adjusted odds ratio; ART, antiretroviral therapy; CI, confidence interval; ATI, antiretroviral therapy interruption; CNY, Chinese New Year; PLHIV, people living with HIV.

^a^ 7 missing.

### 3.6 Attitude towards the government responses to ATI

Among all participants, 67.1% was aware of the “Notice on Ensuring Free Antiviral Therapy Drugs for Stranded People Living with HIV” issued by the Chinese Center for AIDS/STD Control and Prevention (Table S2). The majority of participants became aware of the Notice through community‐centred social media (82.2% from Li Hui Shi Kong, 35.1% from a friend via WeChat and 18.7% from Bai Hua Lin), and 59.1% was satisfied or very satisfied with the government responses to ATI.

## 4 Discussion

We found many PLHIV in China were at risk of ATI and nearly 3% had already experienced an interruption in ART during the COVID‐19 outbreak. PLHIV were more likely to have experienced or been at risk of ATI if they had previous interruptions in ART, travelled away from where they typically receive HIV care or lived in an area that implemented strict COVID‐19 prevention and control measures. The majority of PLHIV who experienced ATI identified citywide lockdowns and travel restrictions as a significant barrier to accessing ART. Obtaining additional ART from HIV clinics other than one’s site of primary HIV care was prohibitively cumbersome, and PLHIV worried that actively seeking ART refills from new sources would disclose their HIV status. Many PLHIV resorted to seeking ART from peers or CBOs, often unsuccessfully. Few previous studies have investigated the impact of COVID‐19 on HIV care, and our findings suggest an urgent need for interventions to maintain access to ART during public health emergencies.

Among the 5084 PLHIV who responded to our online survey, more than one‐third were at risk of ATI, including 2.7% who had experienced ART and 18.0% who had fewer than 10 days of ART on hand without an avenue to obtain additional medication, the continuing traffic control might exacerbate the risk of ATI. These findings are similar to a smaller online survey of PLHIV in China conducted in February 2020 in which 33% of respondents reported having insufficient stores of ART given ongoing travel restrictions [13]. The potential for COVID‐19 to have a widespread impact on the delivery of effective healthcare and access to HIV treatment for PLHIV has been highlighted in several recent publications [16, 17]. Our findings suggest these concerns are well‐founded, and even in the early stages of this evolving public health emergency PLHIV are beginning to experience suboptimal HIV treatment outcomes. Little has been published on ATI during COVID‐19 outside of China. As COVID‐19 develops into a global pandemic, further research is needed to better quantify and characterize barriers to ART access in high‐, middle‐ and low‐income settings.

Our results suggest travel away from primary sites of HIV care and COVID‐19 control measures, particularly citywide lockdowns and travel restrictions, were significant barriers to obtain ART during the COVID‐19 outbreak. In China, the central government fully subsidizes ART for nearly 780,000 PLHIV [18]. ART is distributed through designated clinics in the patient’s registered city of residence. The system requires patients to collect ART in person on a monthly or quarterly basis. On rare occasions, patients may arrange to have medications posted to them, but this is done at the discretion of the prescribing physician. After 23 January 2020 many Chinese cities implemented strict quarantine measures. Roads in many villages and residential communities were closed. The COVID‐19 outbreak coincided with the Chinese New Year holiday period (10 January to 17 February 2020) when hundreds of millions of people travel to visit family. Our survey showed two in every five PLHIV respondents travelled away from the city or village where they receive primary HIV care, and nearly a quarter of respondents reported they were stranded away from home as a result of lockdown measures. Hence, it is important for regions where many PLHIV would flow in to adjust their ART stock and increase the amount of ART that PLHIV could obtain at a time. More than 80% of all survey respondents reported citywide lockdowns and travel restrictions were a significant risk factor for ATI. As shelter at home, measures are rolled out internationally, special consideration should be given to the impact these measures may have on access to HIV care.

PLHIV at risk of ATI resorted to many sources to obtain additional ART, including requesting their designated HIV clinic deliver ART by post, obtaining ART from local HIV clinics when stranded away from home, and borrowing ART from other PLHIV or CBOs. Only one‐third of these attempts were successful. The most frequently reported barrier to obtaining ART during the COVID‐19 was fear of disclosing one’s HIV status, and fewer than 5% of those at risk of ATI applied for an exemption from travel restrictions to present to an HIV clinic. In contrast, nearly half of those at risk of ATI pretended to have a disease other than HIV so that they were permitted to travel to a hospital or clinic for evaluation and requested ART. Additionally, most PLHIV at risk of ATI reported cumbersome administrative procedures impeded their ability to obtain additional ART from the government HIV clinics. Together these results suggest that simply liberalizing restrictions on the distribution of ART from the government clinics will not adequately address barriers to obtaining HIV care in the face of social disruption from COVID‐19. Although the policy was actually beneficial to stranded PLHIV who have successfully obtained ART and might enhance PLHIV’s confidence in taking the initiative to obtain ART, more effective interventions are needed to guide PLHIV through the process of obtaining ART from new sources while still protecting privacy. Peer navigators may be useful [19]. Home delivery of ART through the post may help PLHIV obtain ART despite travel restrictions and minimize the risk of unintended disclosure of HIV status. However, COVID‐19 has caused significant disruptions to postal services in China, which may explain why receipt of ART through the post was associated with the risk of ATI in our study. Posting ART is an effective way to obtain ART when courier services operate smoothly in public health emergencies. Otherwise, home delivery of ART should be considered [20, 21, 22, 24]. Countries should plan according to their own delivery system and PLHIV’s actual needs.

We identified several correlates of ATI that suggest certain subpopulations of PLHIV may be at higher risk of interruption in ART during COVID‐19. PLHIV who had experienced ATI prior to the COVID‐19 outbreak were eight times more likely to experience ATI during the COVID‐19 outbreak compared to PLHIV without the history of ATI. PLHIV who have difficulty adhering to ART are a vulnerable group requiring special care and support, particularly because they are at higher risk of HIV‐associated mortality and contributing to HIV transmission [24]. ATI was also more common among farmers and people living in rural areas. The government‐operated HIV clinics in China are clustered in urban areas, and PLHIV living in the Chinese countryside faced additional barriers to obtaining ART prior to the COVID‐19 outbreak. Our results suggest that social disruption from COVID‐19 disproportionally impacts PLHIV who were already at higher risk of poor ART adherence.

International organizations and local CBOs serving PLHIV have played an important role in maintaining HIV services in China during the COVID‐19 outbreak. CBOs across China mobilized volunteers to collect ART from clinics and deliver them to PLHIV facing ATI. Li Hui Shi Kong, a large CBO, has delivered ART to over 2000 PLHIV during the COVID‐19 outbreak, and Wuhan TongZhi Center, a CBO serving MSM in Wuhan, has delivered ART to over 2600 PLHIV at the centre of the COVID‐19 epidemic [17]. CBOs have also played an important role in responding to COVID‐19 in other low‐ and middle‐income countries. In the Philippines, CBOs, including Network Plus Philippines, Pinoy Plus Advocacy Pilipinas, the Red Whistle and TLF Share Collective, worked together to implement new guidelines issued by the Department of Health [23]. These guidelines directed local authorities to ensure that PLHIV could collect ART at any local HIV clinic and encouraged the use of courier services for home delivery of medications. These successes suggest health authorities across the globe should collaborate with CBOs to reach out to PLHIV and engage them in HIV care services. Online platforms and social media can be used to disseminate information to PLHIV, including frequently updated lists of sites where ART can be obtained.

There are several limitations to our study. First, the cross‐sectional design of our study prevents us from making firm conclusions about the causes of ATI, and we can only report associations between ATI and a narrow set of self‐reported variables. Second, we defined ATI as not taking ART for one day or more. The definition was relatively loose compared to other studies which defined ATI as not taking ART for at least two days [24], and might limit the generalizability of the conclusion. However, our survey was conducted in the midst of strict traffic control for COVID‐19, PLHIV who did not take ARV for one day during this period were very likely to experience continued ATI. Third, we used convenience sampling, and consequently, those who participated in our study may not be representative of PLHIV in China overall. Participants in our study were mostly younger males. In China, nearly 60% of newly reported PLHIV were 20 to 39 years old [25, 26]. Males outnumber females in newly reported HIV cases in large cities. For example the male/female ratio of newly diagnosed HIV cases was 14:1 in Beijing and 9:1 in Shenzhen [27, 28]. MSM were over‐represented among survey respondents, which are likely the consequence of recruiting through social media accounts popular with MSM who tend to live in urban areas, though MSM accounted for less than a quarter of newly reported HIV cases nationwide in 2019 [29]. Compared to sexual transmission, injecting drug use only accounts for a very small fraction of participants. The national HIV prevalence among people who used drugs declined from 7.5% in 2005 to 2.0% in 2018 [30]. Our research only lasted 12 days on the Internet and we did not recruit people who injected drugs during that short time. Contacting PLHIV who use drugs may require the help of local methadone clinics or related organizations, however, inconveniences caused by COVID‐19 increased the difficulty of such efforts. PLHIV willing to complete our online survey may systematically differ from those unwilling or unable to participate in online research. Fourth, all information in this study was self‐reported, and is thus subject to significant risk of bias. Fifth, PLHIV who acknowledged previous ATI maybe more likely to report ATI or risk of ATI during COVID‐19.

## 5 Conclusions

This is one of the first nationwide studies describing the risk of ATI among PLHIV during the COVID‐19 outbreak. Our study identified correlates and self‐reported risk factors for ATI. We found a significant proportion of PLHIV in China was at risk of ATI during the COVID‐19 outbreak, and many had already experienced interruptions in HIV care although PLHIV at risk of ATI had sought help from various sources. Additional interventions and public health measures are needed to secure continued access to ART during public health emergencies. As COVID‐19 spreads internationally and has developed into a global pandemic, other countries may be able to learn from the Chinese experience to better protect the health of PLHIV. Collaborations between CBOs and public health authorities to expand access to home deliveries of ART may be particularly helpful in preventing ATI in the face of citywide lockdowns, travel restrictions and social disruption from COVID‐19. Countries and regions that are still challenged by the COVID‐19 outbreak are suggested to draw experiences from strategies adopted by both the PLHIV community, community‐based organizations and health authorities to mitigate the impact of ATI in China and implement contextualized strategies in their own settings.

## Competing Interests

All authors declare no conflict of interest. The views or opinions expressed in this paper are those of the authors and not of UNAIDS.

## Authors’ Contributions

HZ and HL conceived and designed the study in consultation with the other authors. HZ, HL and YS designed the questionnaire in consultation with XM, YA, FX, YW, HQ and YC. YS obtained data, and, together with HZ, conceived the analysis and presentation. YS and GL contributed to statistical analysis and interpretation of data. WG, TF, HQ and YC assisted with data analysis. All authors contributed to the interpretation of data and study findings. YS, GL, TF and HZ drafted the manuscript with all authors critically reviewing the paper. All authors have read and approved the final report.

## Funding

This study was supported by the Natural Science Foundation of China Excellent Young Scientists Fund [82022064], the Natural Science Foundation of China International/Regional Research Collaboration Project [72061137001], Natural Science Foundation of China Young Scientist Fund [81703278], the Australian National Health and Medical Research Commission (NHMRC) Early Career Fellowship (grant number APP1092621), the National Science and Technology Major Project of China [2018ZX10721102], the Sanming Project of Medicine in Shenzhen [SZSM201811071], the High Level Project of Medicine in Longhua, Shenzhen [HLPM201907020105], and the National Key Research and Development Program of China [2020YFC0840900]. All funding parties did not have any role in the design of the study or in the explanation of the data.

## Supporting information


**Table S1.** Sources of additional ART among PLHIV at risk of ATI before and during the COVID‐19 outbreak in China

**Table S2.** Knowledge and attitude towards ATI‐related policies
