# Supplementary material for: Antiretroviral treatment interruption among people living with HIV during COVID‐19 outbreak in China: a nationwide cross‐sectional study
Source: J Int AIDS Soc. 2020 Nov 1;23(11):e25637. doi: 10.1002/jia2.25637 (PMC7645858; doi:10.1002/jia2.25637)
Supplement: Supplementary file 1 — Table S1. Sources of additional ART among PLHIV at risk of ATI before and during the COVID‐19 outbreak in China [file JIA2-23-e25637-s001.docx]

**Supplementary Table S1. Sources of additional ART among PLHIV at risk of ATI before and during the COVID-19 outbreak in China**

| **Characteristics** | | **n (%)** |
| --- | --- | --- |
| **During the COVID-19 outbreak** | |  |
| Attempted source of ART | |  |
|  | Requested ART be posted by one’s primary HIV clinic | 844 (47.36) |
|  | Requested ART from a nearby HIV clinic other than one’s primary HIV clinic | 468 (26.26) |
|  | Attempted to borrow ART from other PLHIV | 550 (30.86) |
|  | Attempted to obtain ART from CBOs serving PLHIV | 380 (21.32) |
|  | Attempted to purchase from a pharmacy | 75 (4.21) |
|  | Attempted to purchase through a surrogate | 105 (5.89) |
|  | Other | 346 (19.42) |
| Success in requests for ART to be posted by one’s primary HIV clinic | |  |
|  | ART posted and received | 195 (23.10) |
|  | ART posted but not yet received | 108 (12.80) |
|  | Request for ART to be posted was denied | 541 (64.10) |
| No. days of ART obtained from a nearby HIV clinic other than one’s primary HIV clinic | |  |
|  | ≤10 days | 8 (1.71) |
|  | 11-15 days | 6 (1.28) |
|  | 16-30 days | 131 (27.99) |
|  | Failed to obtain additional ART | 323 (69.02) |
| No. days of ART obtained from other PLHIV | |  |
|  | ≤10 days | 103 (18.73) |
|  | 11-20 days | 44 (8.00) |
|  | ≥21 days | 65 (11.82) |
|  | Failed to barrow ART from other PLHIV | 338 (61.45) |
| No. days of ART obtained from CBOs | |  |
|  | ≤10 days | 31 (8.16) |
|  | 11-20 days | 14 (3.68) |
|  | ≥21 days | 70 (18.42) |
|  | Failed to obtain ART from CBOs | 265 (69.74) |
| **Sources of additional ART when at risk of ATI prior to COVID-19 ^a^** | |  |
|  | Requested ART be posted by one’s primary HIV clinic | 87 (37.50) |
|  | Requested ART from a nearby HIV clinic other than one’s primary HIV clinic | 31 (13.36) |
|  | Attempted to borrow ART from other PLHIV | 114 (49.14) |
|  | Attempted to obtain ART from CBOs serving PLHIV | 43 (18.53) |
|  | Attempted to purchase from a pharmacy | 17 (7.33) |
|  | Attempted to purchase through a surrogate | 18 (7.76) |
|  | Other | 41 (17.67) |

Notes: ART, antiretroviral therapy; PLHIV, people living with HIV; ATI, antiretroviral therapy interruption; CBOs, community-based organizations

^a^ Observations in this variable represented participants who couldn’t obtain antiviral medications in time in history.
