# Supplementary material for: Antiretroviral treatment interruption among people living with HIV during COVID‐19 outbreak in China: a nationwide cross‐sectional study
Source: J Int AIDS Soc. 2020 Nov 1;23(11):e25637. doi: 10.1002/jia2.25637 (PMC7645858; doi:10.1002/jia2.25637)
Supplement: Supplementary file 2 — Table S2. Knowledge and attitude towards ATI‐related policies [file JIA2-23-e25637-s002.docx]

**Supplementary Table S2.** **Knowledge and attitude towards ATI-related policies**

| **Characteristics** | | **Total (N=5084)**  **n (%)** | **No risk of ATI (N=3302)**  **n (%)** | **Threatened but resolved risk of ATI (N=730)**  **n (%)** | **Risk of imminent ATI (N=917)**  **n (%)** | **Experienced ATI (N=135)**  **n (%)** | ***P* value** |
| --- | --- | --- | --- | --- | --- | --- | --- |
| Aware of the “Notice on Ensuring Free Antiviral Therapy Drugs for Stranded People Living with HIV” | | | | | | | **0.004** |
|  | Yes | 3413 (67.13) | 2227 (67.44) | 520 (71.23) | 588 (64.12) | 78 (57.78) |  |
|  | No | 1097 (21.58) | 719 (21.77) | 138 (18.90) | 201 (21.92) | 39 (28.89) |  |
|  | Not sure | 574 (11.29) | 356 (10.78) | 72 (9.86) | 128 (13.96) | 18 (13.33) |  |
| Source of information about the “Notice on Ensuring Free Antiviral Therapy Drugs for Stranded People Living with HIV” | | | | | | |  |
|  | Message from a friend on WeChat | 1199 (35.13) | 721 (32.38) | 227 (43.65) | 223 (37.93) | 28 (35.90) | **<0.001** |
|  | Li Hui Shi Kong social media post | 2807 (82.24) | 1874 (84.15) | 419 (80.58) | 459 (78.06) | 55 (70.51) | **<0.001** |
|  | Bai Hua Lin social media post | 637 (18.66) | 406 (18.23) | 110 (21.15) | 107 (18.20) | 14 (17.95) | 0.474 |
|  | Chinese Center for AIDS/STD Control and Prevention social media post | 391 (11.46) | 252 (11.32) | 66 (12.69) | 65 (11.05) | 8 (10.26) | 0.794 |
| Satisfaction with government responses to ATI | | | | | | | **<0.001** |
|  | Very satisfied | 1359 (26.73) | 1018 (30.83) | 191 (26.16) | 132 (14.39) | 18 (13.33) |  |
|  | Satisfied | 1643 (32.32) | 1169 (35.40) | 210 (28.77) | 230 (25.08) | 34 (25.19) |  |
|  | Neither satisfied nor unsatisfied | 1576 (31.00) | 928 (28.10) | 237 (32.47) | 362 (39.48) | 49 (36.30) |  |
|  | Unsatisfied | 324 (6.37) | 129 (3.91) | 63 (8.63) | 113 (12.32) | 19 (14.07) |  |
|  | Very unsatisfied | 182 (3.58) | 58 (1.76) | 29 (3.97) | 80 (8.72) | 15 (11.11) |  |

Notes: STD, sexually transmitted diseases; AIDS, acquired immune deficiency syndrome; ATI, antiretroviral therapy interruption

The bold values are statistically significant (*P*<0.05) in Pearson’s Chi squared test
